# Supplementary material for: Badger territoriality maintained despite disturbance of major road construction
Source: PLoS One. 2021 Sep 3;16(9):e0242586. doi: 10.1371/journal.pone.0242586 (PMC8415604; doi:10.1371/journal.pone.0242586)
Supplement: S1 File — This file contains all supporting tables (S1-S16 Tables), text (S1 Text) and figures (S1-S4 Figs). (DOCX) [file pone.0242586.s001.docx]

Badger territoriality maintained despite disturbance of major road construction.

Supplementary Information.

Aoibheann Gaughran^1*^, Enda Mullen^2 ¶^, Teresa MacWhite^3 ¶,^ Peter Maher^3 ¶^,

David J. Kelly^1 &^, Ruth Kelly^4 &^, Margaret Good^1^ and Nicola M. Marples^1^

^1^ Department of Zoology, School of Natural Sciences, Trinity College Dublin, Ireland

^2^ National Parks and Wildlife Service, Department of Housing, Local Government and Heritage, Ireland

^3^ Department of Agriculture, Food and the Marine, Ireland

^4^ Agri-Food and Biosciences Institute, Northern Ireland, UK

* Corresponding author

Email: [gaughra@tcd.ie](mailto:gaughra@tcd.ie) (AG)

^¶^ These authors contributed equally to this work

^&^ These authors contributed equally to this work

MG and NM are Joint Senior Authors

**S1 Table. Badger population metrics reported in the literature, categorised by the authors as either low, medium, high or very high.** (Gaughran, 2018).

| **Country** | **Region** | **Density Category (Gaughran, 2018)** | **sett/ km2** | **inds/ km2** | **adults/ km2** | **inds/ group** | **adults/ group** | **territory size (km2)** | **Population Size** | **Source Reference** |
| --- | --- | --- | --- | --- | --- | --- | --- | --- | --- | --- |
| Albania | - | Low | - | 0.43 | - | - | - | - | 12500 | Griffiths & Thomas (1993) |
| Austria | - | Low | - | 0.36 | - | - | - | - | 30000 | Griffiths & Thomas (1993) |
| Belgium | - | Low | - | 0.11 | - | - | - | - | 35000 | Do Linh San (2002) |
| Belgium | - | Low | - | 0.1 | - | - | - | - | 3000 | Griffiths & Thomas (1993) |
| Britain | - | Medium | 0.25 | 1.39 | - | 5.90 | - | - | 300000 | Wilson et al. (1997) |
| Bulgaria | - | Low | - | 0.35 | - | - | - | - | 35000 | Griffiths & Thomas (1993) |
| Czech | Bucin Forest | Low | 0.18 | 0.98 | - | - | - | - | - | Pelikan & Vackar (1978) |
| Czech | Northern Moravia | Low | 0.02 | 0.12 | - | - | - | - | 1306 | Matyastik & Bicik (1999) |
| Czech | Southern Moravia | Low | - | 0.22 | - | - | - | - | 3366 | Bicik et al. (2000) |
| Czech | - | Low | - | 0.38 | - | - | - | - | 25000 | Griffiths & Thomas (1993) |
| Denmark | - | Low | - | 0.58 | - | - | - | - | 25000 | Griffiths & Thomas (1993) |
| England | Wytham Woods 1996 | Very High | - | 38 | - | 6.50 | - | 0.003 | - | Johnson et al (2002) |
| England | Brighton | Very High | 4.08 | 33 | - | 7.80 | 4.10 | 0.09 | 69 | Huck et al. (2008) |
| England | East Sussex | Medium | 0.48 | 2.88 | 2.88 | 6.00 | - | - | 5285 | Macdonald et al. (1996) |
| England | South Downs | Very High | 3.12 | 19 | - | - | - | - | - | Oestler & Roper (1998) |
| England | Suburban Bristol | High | 1.88 | 7.2 | 4.90 | 3.90 | 2.60 | - | - | Harris & Cresswell (1987) |
| England | Wytham Woods | Very High | 3.77 | 44.33 | 38.00 | - | 7.00 | - | 266 | Macdonald & Newman (2002) |
| England | Wytham Woods | Very High | - | 36.4 | - | - | - | - | - | Macdonald et al. (2009) |
| England | Wytham Woods | Very High | - | 17 | 17.00 | - | - | - | - | Woodroffe et al (1993) |
| England | Wytham Woods 2014 | Very High | 3.83 | 40.8 | 33.33 | 10.65 | 8.70 | 0.26 | 245 | Chris Newman, personal communication |
| England | Yorkshire forest/moor | High | 0.9 | 4.9 | - | 5.50 | - | 0.304 | - | Palphramand et al. (2007) |
| England | Bristol City | High | 1.88 | 8.06 | 5.95 | 3.30 | - | 0.3128 | - | Cresswell & Harris (1988) |
| England | Woodchester Park | Very High | 2.92 | 20 | 13.52 | 6.85 | 4.63 | 0.34 | - | Cheeseman et al. (1987) |
| England | Wytham Woods 1988 | Very High | - | 19.5 | 19.50 | - | 7.70 | 0.40 | - | da Silva (1989) |
| England | Itchen | Very High | - | 12.3 | - | 5.00 | 5.00 | 0.41 | - | Packham (1983) |
| England | Wytham Woods 1982 | High | 0.93 | 3.48 | - | 6.80 | 4.30 | 0.521 | - | Hofer (1988) |
| England | Wytham Woods (mean) | Very High | 2.26 | 17.35 | - | 5.80 | - | 0.6 | - | Johnson et al (2002) |
| England | Wytham Woods 1972-75 | Very High | 2.16 | 15.17 | - | 7.00 | - | 0.87 | 91 | Kruuk (1978) |
| England | Staffordshire | High | 0.96 | 8.64 | - | 6.40 | - | 1.04 | - | Cheeseman et al. (1985) |
| England | Woodchester Park 1 | Very High | 4.55 | 22 | 19.70 | 4.80 | 4.30 | 0.22 | - | Cheeseman et al (1981) |
| England | Woodchester Park 2 | Very High | 4.03 | 30.7 | 19.40 | 7.60 | 5.80 | 0.248 | - | Cheeseman et al (1981) |
| England | Woodchester Park 1993 | Very High | 2.87 | 36.03 | 25.30 | 12.50 | 8.80 | 0.35 | - | Rogers et al (1997) |
| England | Staffordshire | High | - | 6.2 | - | - | - | - | - | Cheeseman et al (1985) |
| England | Suburban Bristol | High | - | 5.5 | - | - | - | - | - | Cheeseman et al (1988) |
| England | Woodchester Park | High | - | 9 | - | - | - | - | - | Cheeseman et al (1988) |
| England | Avon | High | 1.36 | 7.75 | 4.90 | 5.70 | 3.60 | 0.737 | - | Cheeseman et al (1981) |
| England | Cornwall | High | 1.34 | 6.5 | 4.70 | 4.80 | 3.30 | 0.747 | - | Cheeseman et al (1981) |
| Estonia | - | Low | - | 0.04 | - | - | - | - | 2000 | Griffiths & Thomas (1993) |
| Finland | - | Low | - | 0.32 | - | - | - | - | 70000 | Griffiths & Thomas (1993) |
| Finland | South East Finland | Low | - | 0.24 | - | - | - | - | 1470 | Kauhala et al (2006) |
| France | - | Low | - | 0.15 | - | - | - | - | 80000 | Griffiths & Thomas (1993) |
| France | - | Medium | - | 1.6 | - | - | - | 25 | - | Mouches (1982) |
| Germany | - | Low | - | 0.29 | - | - | - | - | 72000 | Griffiths & Thomas (1993) |
| Germany | - | Low | - | 0.8 | - | - | - | - | - | Keuling et al (2011) |
| Germany | - | Low | - | 0.4 | - | - | - | - | 142000 | Keuling pers. comm. in Roper (2010) |
| Hungary | - | Low | - | 0.21 | - | - | - | - | 20000 | Griffiths & Thomas (1993) |
| Ireland (Rep) | Little Island | Very High | 6.5 | 34.15 | 27.50 | - | - | 0.2061 | - | Sleeman et al. (2010) |
| Ireland (Rep) | Kilmurry, Cork 1990 | Medium | 0.6 | 2.9 | - | - | 4.50 | 0.70 | - | Sleeman & Mulcahy (2005) |
| Ireland (Rep) | Offaly | High | 0.65 | 4.4 | 4.00 | - | 4.00 | 0.874 | - | O’Corry Crowe, Eves & Hayden (1993) |
| Ireland (Rep) | Cork | Medium | 0.62 | 1.8 | - | 4.45 | 4.45 | - | - | Sleeman et al. (2009) |
| Ireland (Rep) | Donegal | Medium | 0.44 | 1.06 | - | 3.58 | 3.58 | - | - | Sleeman et al. (2009) |
| Ireland (Rep) | Four Areas (mean) | Medium | 0.51 | 1.14 | - | 3.94 | 3.94 | - | 84000 | Sleeman et al. (2009) |
| Ireland (Rep) | Kilkenny | Medium | 0.51 | 1.08 | - | 4.32 | 4.32 | - | - | Sleeman et al. (2009) |
| Ireland (Rep) | Kilmurry, Cork 1993 | Medium | 0.1 | 1.2 | - | - | 2.25 | - | - | Sleeman & Mulcahy (2005) |
| Ireland (Rep) | Monaghan | Low | 0.48 | 0.85 | - | 3.41 | 3.41 | - | - | Sleeman et al. (2009) |
| Ireland (Rep) | Offaly | Medium | 1.54 | 2.9 | - | 3.05 | - | - | - | Eves (1999) |
| Ireland (Rep) | Wicklow | Medium | 0.275 | 1.1 | - | - | - | 1.9 | - | Gaughran (2018) |
| Ireland (Rep) | - | Medium | 0.5 | 2.8 | - | 5.90 | - | - | 195000 | Smal (1995) |
| Ireland (Rep) | Meath | Medium | 0.71 | 2 | 0.80 | 4.17 | 1.67 | - |  | Elliot et al. (2015) |
| Italy | Maremma NP | Medium | - | 1 | - | 1.00 | - | 0.73 | - | Pigozzi (1987) |
| Kazakhstan | Dzhungarskii Alatau | Medium | 0.65 | 1.52 | - | - | - | - | - | Lobachev (1976) |
| Latvia | Slitere Reserve | Low | 0.11 | 0.3 | - | - | - | - | - | Zoss (1992) |
| Lithuania | - | Low | - | 0.05 | - | - | - | - | 3000 | Griffiths & Thomas (1993) |
| Luxembourg | Eppeldorf & Medernach | High | 0.99 | 4.83 | - | 4.75 | - | - | - | Scheppers et al. (2007) |
| Luxembourg | - | Low | 0.17 | 0.78 | 0.44 | 4.60 | 2.60 | - | 2010 | Schley et al (2004) |
| N. Ireland | Castleward, Down | Very High | 2.27 | 17.5 | 11.90 | 9.30 | 6.30 | 0.504 | - | Feore & Montgomery (1999) |
| N. Ireland | Down | Medium | 0.95 | 2.35 | 1.05 | 3.60 | 1.79 | - | - | Sadlier & Montgomery (2004) |
| N. Ireland | - | Medium | 0.56 | 2.51 | - | 4.16 | - | - | 34100 | Reid et al. (2011) |
| N. Ireland | Katesbridge, Down | Medium | 0.79 | 1.6 | - | 2.00 | 2.00 | 1.274 | - | Feore & Montgomery (1999) |
| N. Ireland | 3 Areas Mean | Very High | 1.13 | 10.4 | 9.03 | 4.60 | 3.43 | 1.7427 | 38000 | Feore & Montgomery (1999) |
| N. Ireland | Glenwhirry, Antrim | Low | 0.34 | 0.86 | 0.72 | 2.50 | 2.00 | 3.45 | - | Feore & Montgomery (1999) |
| Netherlands | - | Low | - | 0.06 | - | - | - | - | 2200 | Griffiths & Thomas (1993) |
| Netherlands | - | Medium | - | 1 | - | - | - | - | - | Wijngaarden & Peppel (1964) |
| Netherlands | Utrecht | Low | 0.12 | 0.46 | 0.33 | - | 2.30 | 1.12 | - | Van Apeldoorn et al. (2006) |
| Norway | Malvik | Low | 0.07 | 0.16 | - | 2.30 | 2.30 | - | - | Brøseth et al. (1997) |
| Norway | - | Low | - | 0.23 | - | - | - | - | 45000 | Griffiths & Thomas (1993) |
| Poland | Bialowieza Forest | Low | 0.04 | 0.16 | - | 3.80 | 3.80 | - | - | Kowalczyk et al. (2003) |
| Poland | Rogow | Low | 0.11 | 0.31 | - | 3.50 | 2.10 | - | - | Goszezyiiski & Skoczyfiska (1996) |
| Poland | Suwalki | Low | 0.1 | 0.59 | 0.36 | 5.80 | 3.50 | - | - | Goszczynski (1999) |
| Poland | - | Low | - | 0.04 | - | - | - | - | 12000 | Griffiths & Thomas (1993) |
| Poland | Bialowieza Forest (Polish) | Low | 0.04 | 0.16 | - | 3.80 | - | 17 | - | Kowalczyk et al. (2000) |
| Poland | Bialowieza Forest (whole) | Low | 0.02 | 0.08 | - | 3.80 | - | 22 | - | Kowalczyk et al. (2000) |
| Poland | Bialowieza Forest (Belarssian 1946-51) | Low | 0.03 | 0.13 | - | 3.80 | 3.80 | 32 | - | Kowalczyk et al. (2000) |
| Poland | Bialowieza Forest (Belarssian 1979-99) | Low | 0.02 | 0.06 | - | 3.80 | 3.80 | 32 | - | Kowalczyk et al. (2000) |
| Portugal | Sierra de Grandola | Low | - | 0.42 | - | 7.00 | 4.00 | 4.46 | - | Rosalino et al. (2004) |
| Russia | Kivach Reserve | Low | 0.12 | 0.46 | - | - | - | - | - | Ivanter (1973) |
| Scotland | Aviemore | Medium | - | 2.2 | - | - | 3.60 | 1.21 | - | Kruuk & Parish (1987) |
| Scotland | New Deer | High | 0.48 | 5.95 | - | 9.50 | 9.50 | 1.59 | - | Kruuk & Parish (1982) |
| Scotland | Ardnish | Medium | 0.58 | 2.1 | - | 3.50 | 3.50 | 1.73 | - | Kruuk & Parish (1982) |
| Scotland | Aviemore/Speyside | Medium | 0.63 | 2.06 | - | 4.00 | 4.00 | 2.06 | - | Kruuk & Parish (1982) |
| Spain | Monserrat | Medium | - | 1.9 | - | 3.00 | - | 0.908 | - | Molina-Vacas et al. (2009) |
| Spain | Donana NP Coto del Ray | Low | - | 0.85 | - | 4.56 | 2.00 | 4.06 | - | Revilla et al (1999) and Revilla & Palomares (2002) |
| Spain | Collserola Park | Low | - | 0.6 | - | 1.50 | - | 4.16 | - | Molina-Vacas et al. (2009) |
| Spain | Donana NP | Low | - | 0.5 | - | 2.00 | - | 4.22 | - | martin & Delibes (1985) |
| Spain | Donana NP | Low | 0.16 | 0.36 | - | 1.50 | - | 5.25 | - | Rodriguez et al. (1996) |
| Spain | Donana NP | Low | - | 0.28 | - | 2.00 | - | 7.80 | - | Revilla et al. (1999) |
| Spain | Donana NP (mean) | Low | 0.16 | 0.5 | - | 2.30 | - | - | - | In Johnson et al. (2002) |
| Spain | Donana NP Res Biologica | Low | - | 0.28 | 0.23 | 3.33 | 2.00 | 7.80 | - | Revilla et al (1999) and Revilla & Palomares (2002) |
| Sweden | - | Medium | - | 2.8 | 2.80 | - | - | 20 | - | Ahnlund & Lindahl, in Anderson & Trewhella (1985) |
| Sweden | - | Medium | - | 1.35 | - | - | - | - | 350000 | Griffiths & Thomas (1993) |
| Switzerland | BCT Jura Foothills | Medium | 0.42 | 1.8 | 1.04 | 3.80 | 2.20 | 2.12 | - | Do Linh San (2007a) |
| Switzerland | Chaux d'Abel | Medium | 0.1 | 1 | 0.59 | 3.40 | 2.00 | 3.40 | - | Do Linh San (2007b) |
| Switzerland | Berne | Low | - | 0.5 | - | - | - | 3.87 | - | Graf et al. (1996) |
| Switzerland | - | Low | - | 0.18 | - | - | - | - | 7500 | Griffiths & Thomas (1993) |

**S1 Fig. Map of the construction zone.** The road upgrade involved building a new 16km section of motorway (M11) adjacent to the original national road (N11, grey line). The construction zone is enclosed by the red line. It was on average 100m wide (max. 400m), with a total area of 2km2 and a perimeter of 39km.


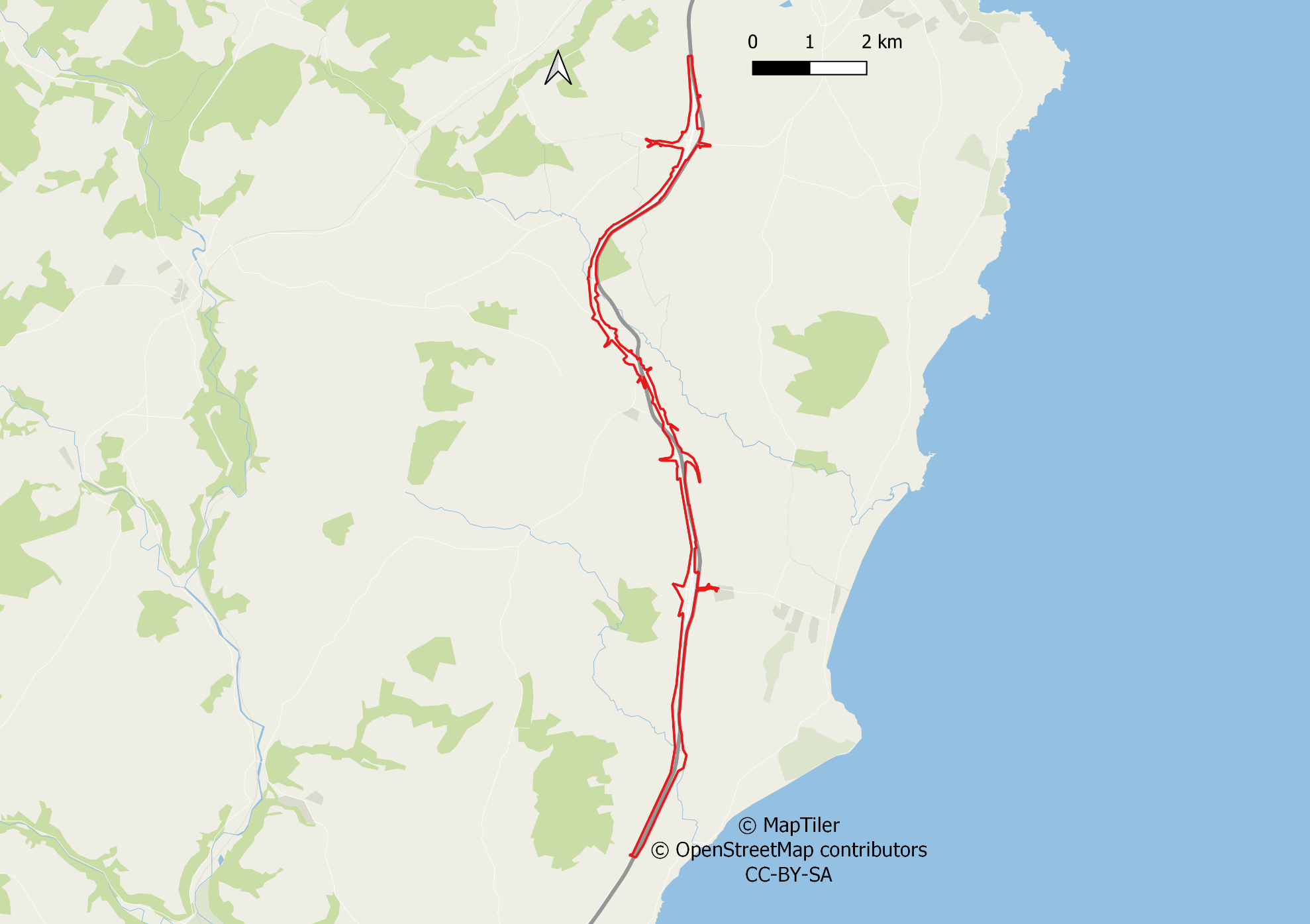


**S2 Table. Number of individual badgers that were categorised as traditional rangers (the focus of this manuscript), super-rangers (Gaughran *et al.*,2018) and dispersers (Gaughran *et al.*,2019) during each phase of the road construction process.**

|  | **Traditional Ranger** | **Super-Ranger** | **Disperser** |
| --- | --- | --- | --- |
| **Before Road Construction** | 39 | 9 | 7 |
| **During Road Construction** | 36 | 8 | 5 |
| **After Road Construction** | 34 | 7 | 4 |

**S2 Fig. Comparison of 2010 social group boundaries (unhatched polygons) with boundaries as suggested by earlier bait marking studies (hatched polygons)**. Bait-marking had suggested territories that were much smaller than those quickly revealed by GPS tracking data.

**
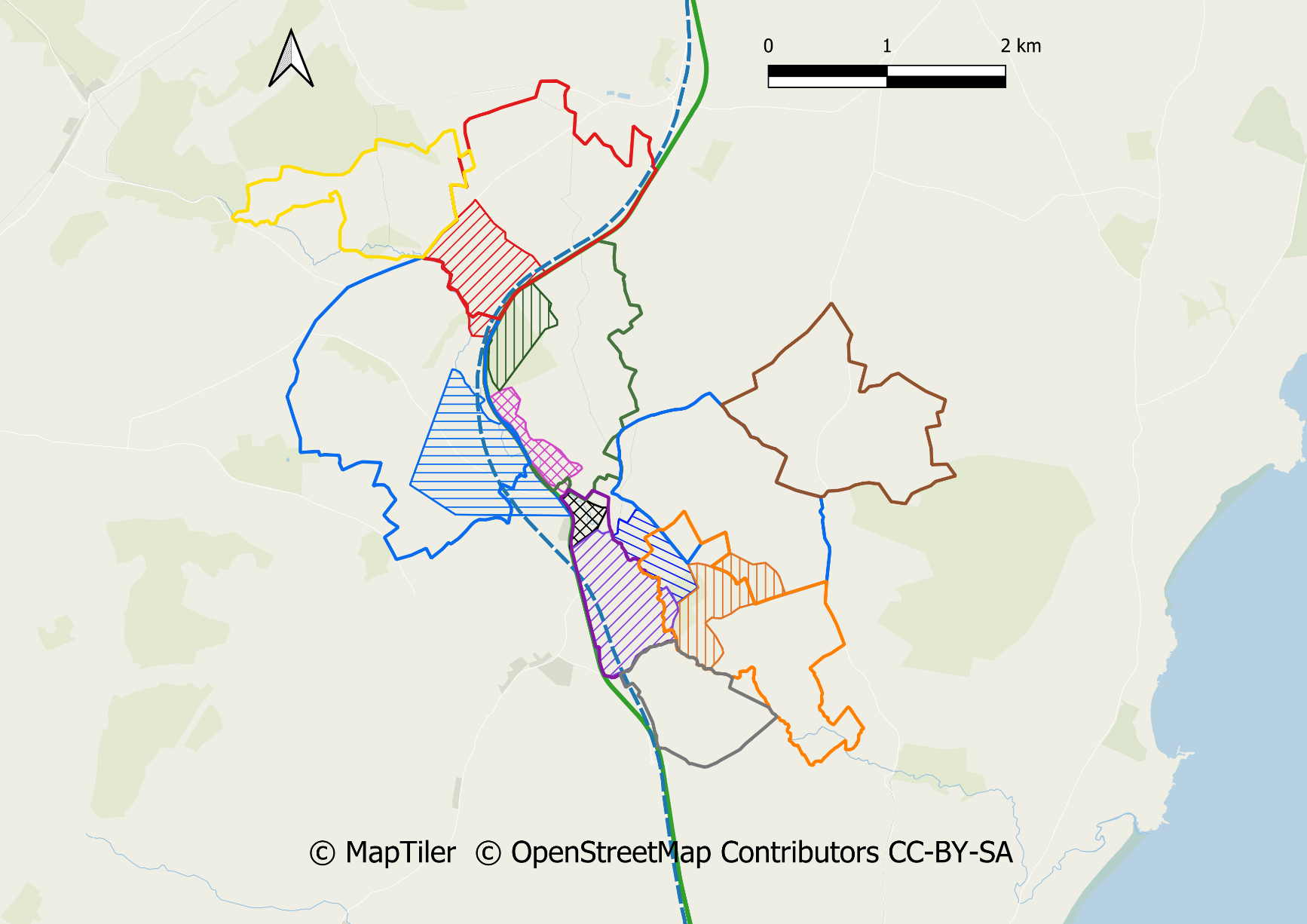
**

**S1** **Text.** **Procedure for mapping geographical territory boundaries.**

In order to map realistic territory boundaries and to decide objectively which GPS locations constituted ETEs, summer MCPs were plotted in ArcMap along with all of the GPS locations. A ‘geographical territory boundary’ was digitised based on the location of a summer MCP polygon, the real linear landscape features in the study area (roads, hedgerows, rivers and observed badger-paths) and the GPS locations of the relevant social group. Polygon shapefiles were created by following the MCP boundary line as closely as possible to the linear feature that was nearest to, and that encompassed the GPS locations. Where the MCP line was equidistant between two linear features *e.g.* through the middle of a field, the linear feature that was closest to the location of the GPS fixes was followed. Where linear features coincided with the outermost GPS fixes unambiguously, these linear features were followed. Where the MCP line was convex and the pattern of the GPS fixes concave, the linear features that were closest to the cluster of GPS fixes for the social group in question were followed. This procedure was carried out for each social group in each calendar year.

**S3 Fig. Social group boundary maps for each year of the study.** The final figure is a composite map for all years combined.

**
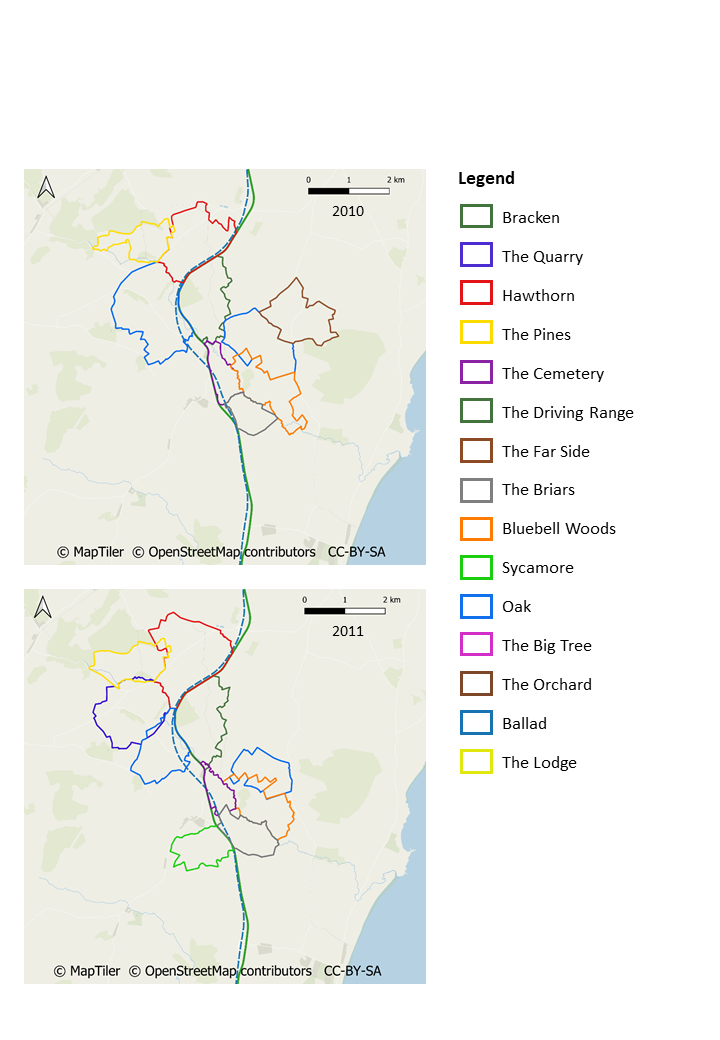
**

2013

**
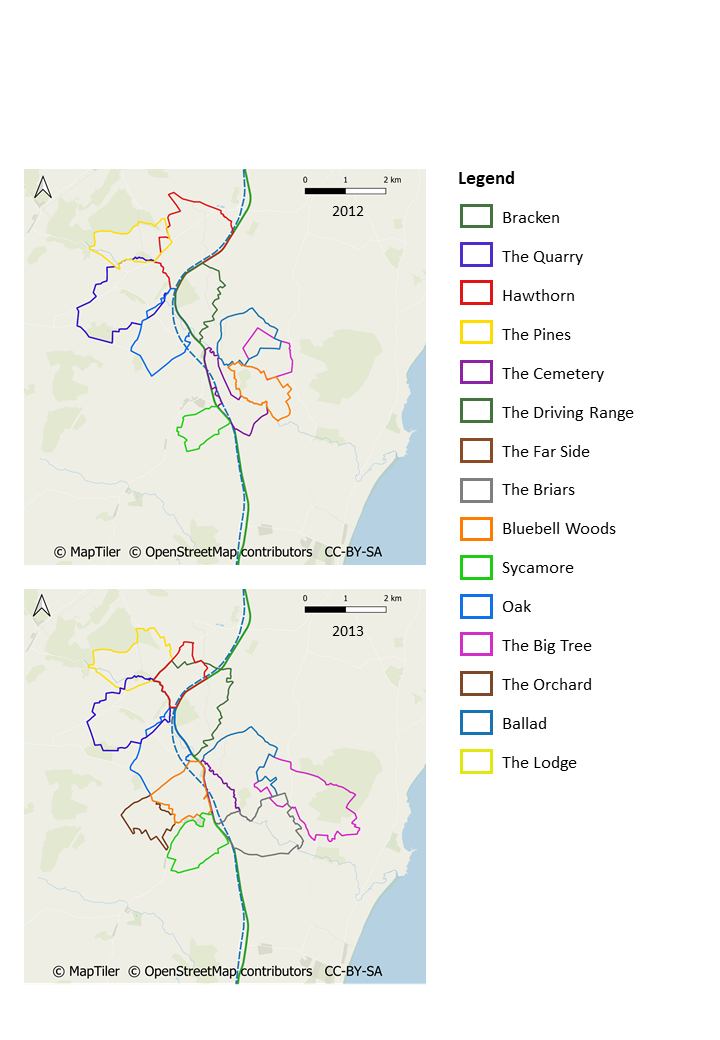
**

**
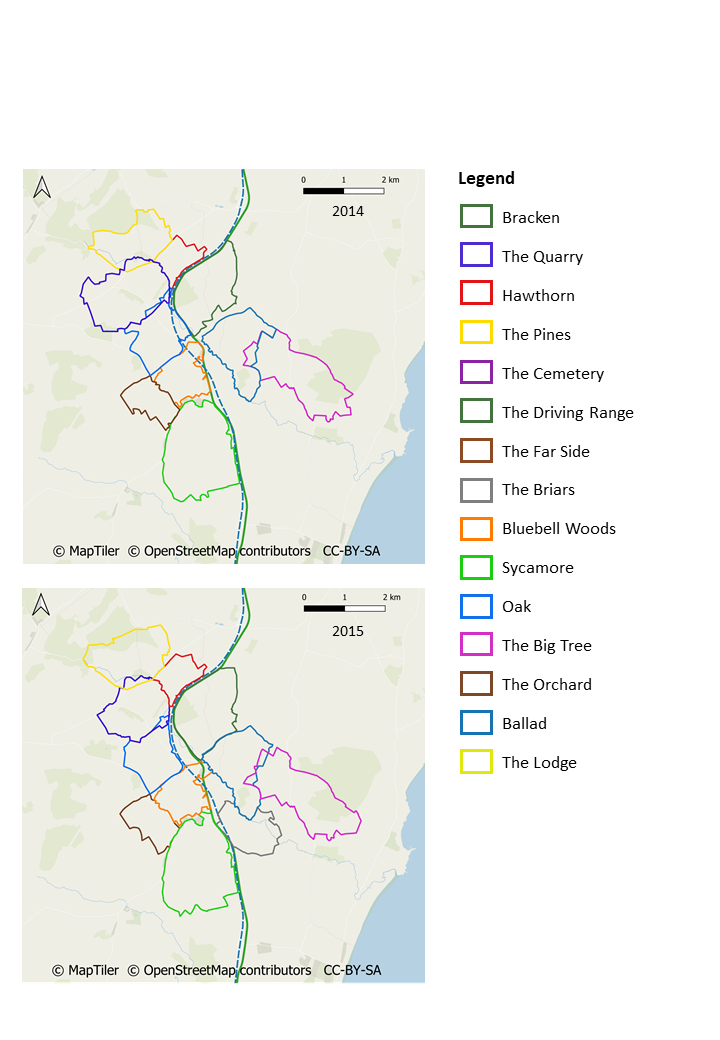
**

**
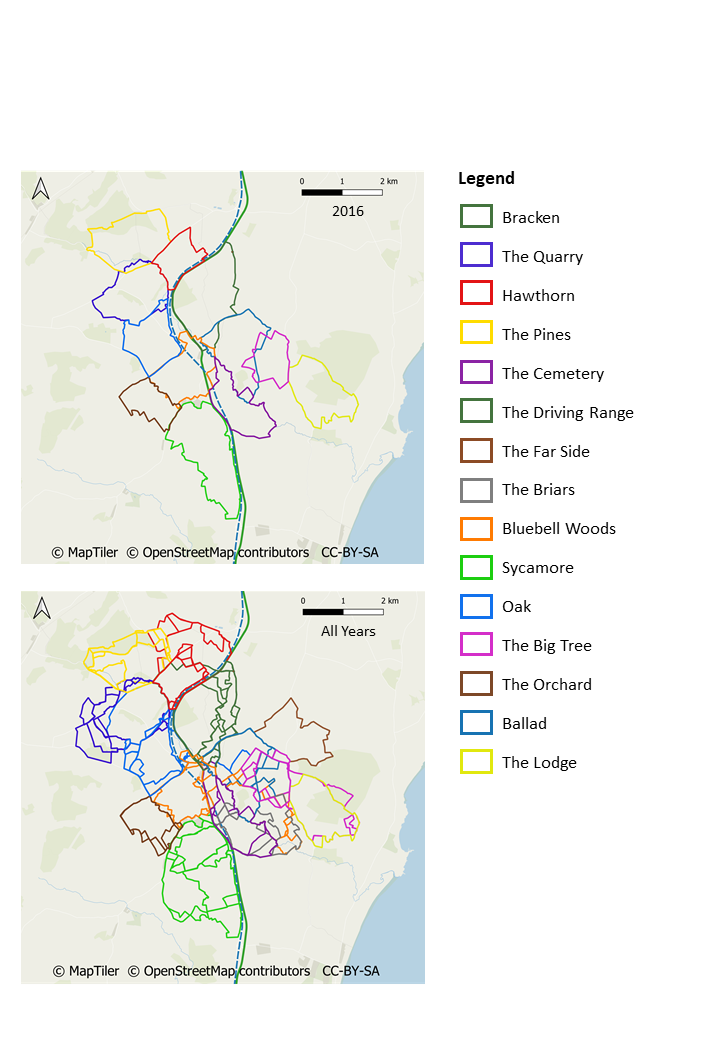
**

**S3 Table. Nightly distance model selection.** Comparison of top models returned at ΔAICc2.

| **Model** | **Components** | **df** | **logLik** | **AICc** | **Delta** | **Weight** |  |
| --- | --- | --- | --- | --- | --- | --- | --- |
| **Top Model** | 123457 | 26 | -1304.61 | 2661.30 | 0.00 | 0.72 |  |
| **Second Best Model** | 1234567 | 28 | -1303.54 | 2663.17 | 1.87 | 0.28 |  |
| **Number of models by component** | | |  |  |  |  |  |
| **Components** | 1  Adjacent | 2  sex | 3  cohort | 4  month | 5  roadworks | 6  adjacent:  roadworks | 7  sex:  cohort |
| **No. Models** | 2 | 2 | 2 | 2 | 2 | 1 | 2 |

**S4 Table. Home range size model selection.** Comparison of top models returned at ΔAICc2.

| **Model** | **Components** | **df** | **logLik** | **AICc** | **Delta** | **Weight** |
| --- | --- | --- | --- | --- | --- | --- |
| **Top Model** | 2345 | 23 | -33.05 | 113.38 | 0.00 | 0.72 |
| **Second Best Model** | 12345 | 24 | -.32.92 | 115.22 | 1.85 | 0.28 |
| **Number of models by component** | | |  |  |  |  |
| **Components** | 1  adjacent | 2  sex | 3  age cohort | 4  month | 5  sex:  age cohort |  |
| **No. Models** | 1 | 2 | 2 | 2 | 2 |  |

**S5 Table. ETE distance model selection.** Comparison of top models returned at ΔAICc2.

| **Model** | **Components** | **df** | **logLik** | **AICc** | **Delta** | **Weight** |
| --- | --- | --- | --- | --- | --- | --- |
| **Top Model** | 2345 | 23 | -26667.84 | 53385.98 | 0.00 | 0.57 |
| **Second-Best Model** | 12345 | 24 | -26669.76 | 53387.84 | 1.86 | 0.22 |
| **Third-Best Model** | 234 | 25 | -26673.86 | 53387.95 | 1.97 | 0.21 |
| **Number of models by component** | | |  |  |  |  |
| **Components** | 1  adjacent | 2  sex | 3  age cohort | 4  month | 5  sex: age cohort |  |
| **No. Models** | 1 | 3 | 3 | 3 | 2 |  |

**S6 Table. Frequency of ETE model selection.** Comparison of top models returned at ΔAICc2.

| **Model** | **Components** | **df** | **logLik** | **AICc** | **Delta** | **Weight** |  |
| --- | --- | --- | --- | --- | --- | --- | --- |
| **Top Model** | 2345 | 22 | -2064.05 | 4173.3 | 0.00 | 0.45 |  |
| **Second-Best Model** | 234 | 21 | -2065.31 | 4173.7 | 0.23 | 0.37 |  |
| **Third-Best Model** | 12345 | 23 | -2063.95 | 4175.2 | 0.93 | 0.18 |  |
| **Number of models by component** | | |  |  |  |  |  |
| **Components** | 1  adjacent | 2  age | 3  month | 4  roadworks | 5  sex | 6  rw: adj |  |
| **No. Models** | 1 | 3 | 3 | 3 | 2 | 0 |  |

**S7 Table. GLMM model specification for analysis of badger movement metrics.** Data transformation, distribution family and explanatory variables specified in the best model for ND, HR, ETE distance and ETE frequency, as well as R package used. Colons indicate interaction terms.

| **Response Variable** | **Transformation** | **Distribution**  **family** | **Explanatory Variables** | **R package** |
| --- | --- | --- | --- | --- |
| ND (m) | BoxCox  (lambda = 0.384) | Gaussian | sex:age cohort  month  adjacency  roadworks | lme4 |
| HR (km^2^) | BoxCox  (lambda = 0.505) | Gaussian | sex:age cohort  month | lme4 |
| ETE (m) | None | Gamma (log) | sex:age cohort  month | lme4 |
| fETE  (proportion, 0-1) | None | Beta binomial (logit) | sex  month  roadworks  cohort | glmmTMB |

**S8** **Table.** **Summary statistics of nightly distance travelled by badgers.** ND (m) mean, SD, median, min, max, 95^th^ percentile and number of observations for the different sexes and age cohorts, for all badgers before, during and after the roadworks, and for all badgers in social groups adjacent or non-adjacent to the roadworks area.

| **Group** | **Mean**  **ND (m)** | **SD ±** | **Median**  **ND (m)** | **Min (m)** | **Max (m)** | **95^th^ percentile** | **No. observations** |
| --- | --- | --- | --- | --- | --- | --- | --- |
| All badgers | 834.7 | 656.5 | 696.7 | 0.2 | 11251.5 | 2056.2 | 18954 |
| Females | 808.2 | 645.4 | 661.7 | 0.2 | 9236.2 | 2010.0 | 9451 |
| Males | 861.0 | 666.4 | 732.3 | 1.5 | 11251.5 | 2094.2 | 9503 |
| Cubs | 343.6 | 302.9 | 264.8 | 2.7 | 2098.5 | 911.6 | 757 |
| Younger adults | 809.1 | 637.3 | 673.1 | 0.2 | 11251.5 | 1972.1 | 7924 |
| Older adults | 873.9 | 660.7 | 748.5 | 1.9 | 10051.6 | 2093.0 | 9246 |
| Aged adults | 1040.4 | 765.7 | 867.2 | 3.9 | 4959.9 | 2523.4 | 1027 |
| Before roadworks | 756.9 | 621.9 | 602.0 | 1.5 | 9236.2 | 1951.4 | 7713 |
| During roadworks | 850.0 | 671.5 | 716.4 | 0.2 | 11251.5 | 2039.1 | 5428 |
| After roadworks | 923.5 | 674.7 | 790.9 | 3.0 | 8611.7 | 2203.3 | 5813 |
| Adjacent | 809.9 | 657.9 | 654.3 | 0.2 | 9236.2 | 2050.7 | 10904 |
| Non-adjacent | 868.2 | 653.2 | 748.0 | 2.6 | 11251.5 | 2064.0 | 8050 |

**S9 Table.** **Summary table of GLMM results for nightly distance travelled.** The results for each factor level of a categorical variable are listed in order and are in comparison to the base-level of that factor. For example, the variable cohort has four factors – cub; young adult, older adult and aged. The results displayed for young adult are in comparison to cub, which is the base-level factor in this case. Similarly, each month is being compared to January, the base-level for that variable.

| GLMM with Gaussian distribution, response variable boxcox transformed (km ^ 0.3838384) | | | | | | |
| --- | --- | --- | --- | --- | --- | --- |
| Number of Observations: 18,954, Groups: name: socgrp, 89; socgrp, 18; year, 7 | | | | | |  |
| *Random Effects* | Variance | Standard Deviation | |  |  |  |
| name: socgrp | 0.0055803 | 0.0747 |  |  |  |  |
| socgrp | 0.0000000 | 00.000 |  |  |  |  |
| Year | 0.0001539 | 0.0129 |  |  |  |  |
| Residual | 0.0663671 | 0.2576 |  |  |  |  |
| *Fixed effects* | Estimate | Std. Error | df | t value | Pr(>\|t\|) |  |
| (Intercept) | 5.65E-01 | 3.16E-02 | 2.59E+02 | 17.913 | <2e-16 | *** |
| Adjacent to RW | -5.02E-02 | 1.73E-02 | 6.80E+01 | -2.904 | 0.004958 | ** |
| Male | 3.76E-02 | 3.16E-02 | 2.47E+02 | 1.188 | 0.235857 |  |
| Younger Adult | 1.04E-01 | 2.69E-02 | 3.22E+02 | 3.852 | 0.000142 | *** |
| Older Adult | 9.79E-02 | 2.77E-02 | 3.00E+02 | 3.535 | 0.000473 | *** |
| Aged Adult | 1.01E-01 | 3.30E-02 | 4.15E+02 | 3.057 | 0.002382 | ** |
| February | 7.30E-02 | 1.51E-02 | 1.89E+04 | 4.849 | 1.25E-06 | *** |
| March | 1.29E-01 | 1.38E-02 | 1.90E+04 | 9.347 | <2e-16 | *** |
| April | 2.11E-01 | 1.32E-02 | 1.89E+04 | 15.913 | <2e-16 | *** |
| May | 2.49E-01 | 1.23E-02 | 1.85E+04 | 20.188 | <2e-16 | *** |
| June | 2.82E-01 | 1.25E-02 | 1.84E+04 | 22.606 | <2e-16 | *** |
| July | 2.28E-01 | 1.26E-02 | 1.43E+04 | 18.036 | <2e-16 | *** |
| August | 2.12E-01 | 1.28E-02 | 1.49E+04 | 16.586 | <2e-16 | *** |
| September | 1.67E-01 | 1.34E-02 | 9.59E+03 | 12.45 | <2e-16 | *** |
| October | 5.10E-02 | 1.31E-02 | 8.43E+03 | 3.882 | 0.000105 | *** |
| November | -7.92E-03 | 1.32E-02 | 7.12E+03 | -0.601 | 0.547964 |  |
| December | -5.22E-02 | 1.43E-02 | 8.21E+03 | -3.655 | 0.000259 | *** |
| During RW | 5.50E-02 | 1.05E-02 | 9.80E+01 | 5.245 | 8.97E-07 | *** |
| After RW | 7.83E-02 | 1.27E-02 | 6.80E+01 | 6.144 | 4.70E-08 | *** |
| Male: Younger Adult | -2.55E-02 | 3.04E-02 | 5.08E+02 | -0.839 | 0.40209 |  |
| Male: Older Adult | 2.87E-02 | 3.21E-02 | 5.08E+02 | 0.893 | 0.37215 |  |
| Male: Aged Adult | 1.09E-02 | 3.95E-02 | 8.46E+02 | 0.277 | 0.781685 |  |

**S10 Table.** **Summary statistics for badger home range size.** HR (km^2^) mean, SD, median, maximum, minimum (m^2^) and number of observations (No. obvs.) for the different sexes and age cohorts, for all badgers before, during and after the roadworks and all badgers in for social groups adjacent or non-adjacent to the roadworks area.

| **Group** | **Mean**  **HR (km^2^)** | **SD ±** | **Median**  **HR (km^2^)** | **Min (m^2^)** | **Max (km^2^)** | **No. Obvs.** |
| --- | --- | --- | --- | --- | --- | --- |
| All badgers | 1.03 | 0.64 | 0.95 | 42 | 3.39 | 890 |
| Females | 0.98 | 0.68 | 0.85 | 4174 | 3.23 | 454 |
| Males | 1.09 | 0.60 | 1.01 | 42 | 3.39 | 436 |
| Cubs | 0.47 | 0.32 | 0.43 | 4174 | 1.27 | 47 |
| Younger adults | 0.98 | 0.61 | 0.93 | 42 | 3.23 | 383 |
| Older adults | 1.11 | 0.65 | 1.04 | 32399 | 3.39 | 325 |
| Aged adults | 1.19 | 0.68 | 0.99 | 41764 | 2.89 | 135 |
| Before roadworks | 0.98 | 0.64 | 0.88 | 42 | 3.23 | 403 |
| During roadworks | 1.06 | 0.68 | 0.98 | 4174 | 3.39 | 260 |
| After roadworks | 1.10 | 0.59 | 1.01 | 32761 | 2.69 | 227 |
| Adjacent | 1.02 | 0.67 | 0.91 | 42 | 3.39 | 511 |
| Non-adjacent | 1.05 | 0.61 | 0.99 | 3652 | 2.71 | 379 |

**S11 Table.** **Summary table of GLMM results for home range size.** The results for each factor level of a categorical variable are listed in order and are in comparison to the base-level of that factor. For example, the variable cohort has four factors – cub; young adult, older adult and aged. The results displayed for young adult are in comparison to cub, which is the base-level factor in this case. Similarly, each month is being compared to January, the base-level for that variable.

| GLMM with Gaussian distribution, response variable boxcox transformed (km ^ 0.5050505) | | | | | | |
| --- | --- | --- | --- | --- | --- | --- |
| Number of Observations: 890, Groups: name: socgrp, 86; socgrp, 18; year, 7 | | | | | |  |
| *Random Effects* | Variance | Standard Deviation | |  |  |  |
| Name: socgrp | 0.037666 | 0.19408 |  |  |  |  |
| Socgrp | 0.009061 | 0.09519 |  |  |  |  |
| Year | 0.004545 | 0.06742 |  |  |  |  |
| Residual | 0.051198 | 0.22627 |  |  |  |  |
| *Fixed effects* | Estimate | Std. Error | df | t value | Pr(>\|t\|) |  |
| (Intercept) | 0.53902 | 0.09212 | 162.2 | 5.851 | 2.62E-08 | *** |
| Male | 0.09589 | 0.09727 | 262.1 | 0.986 | 0.325141 |  |
| Younger adult | 0.17196 | 0.08034 | 292.2 | 2.14 | 0.033158 | * |
| Older adult | 0.1371 | 0.08408 | 268.6 | 1.63 | 0.104168 |  |
| Aged adult | 0.11053 | 0.09319 | 279.2 | 1.186 | 0.236629 |  |
| February | 0.13831 | 0.04206 | 794.1 | 3.288 | 0.001052 | ** |
| March | 0.14378 | 0.04328 | 794.4 | 3.322 | 0.000934 | *** |
| April | 0.15591 | 0.03812 | 810 | 4.09 | 4.74E-05 | *** |
| May | 0.30601 | 0.03752 | 818 | 8.156 | 1.33E-15 | *** |
| June | 0.32003 | 0.03849 | 819.6 | 8.314 | 4.44E-16 | *** |
| July | 0.33811 | 0.03918 | 816.9 | 8.629 | <2.00E-16 | *** |
| August | 0.33141 | 0.04085 | 814.5 | 8.112 | 1.78E-15 | *** |
| September | 0.3389 | 0.04334 | 812.3 | 7.82 | 1.64E-14 | *** |
| October | 0.13692 | 0.03933 | 818.9 | 3.481 | 0.000525 | *** |
| November | 0.06882 | 0.0391 | 815.7 | 1.76 | 0.078812 | . |
| December | -0.0598 | 0.04048 | 817.7 | -1.477 | 0.14 |  |
| Male: Younger Adult | -0.07342 | 0.0968 | 465.1 | -0.759 | 0.448521 |  |
| Male: Older Adult | 0.15038 | 0.10364 | 444.1 | 1.451 | 0.147476 |  |
| Male: Aged Adult | 0.17514 | 0.11216 | 486.1 | 1.562 | 0.119043 |  |

**S12 Table. Summary statistics for extra-territorial excursion distance in badgers.** ETE distance (m) mean, SD, median, minimum, maximum, 95% percentiles and the number of observations (No. obvs.) for the different sexes and age cohorts, for badgers before, during and after the roadworks and for all badgers in social groups adjacent or non-adjacent to the roadworks area. Note re. minima: all GPS locations that were <15m away from the boundary of a territory were excluded, due to the maximum error of a GPS reading.

| **Group** | **Mean**  **ETE (m)** | **SD ±** | **Median**  **ETE (m)** | **Min ETE (m)** | **Max**  **ETE (m)** | **95^th^ Percentile** | **No. Obvs.** |
| --- | --- | --- | --- | --- | --- | --- | --- |
| All badgers | 248.7 | 351.7 | 112.7 | 15 | 4232.1 | 951.7 | 3726 |
| Females | 178.0 | 292.5 | 87.1 | 15 | 4167.6 | 568.8 | 1708 |
| Males | 308.5 | 384.9 | 144.1 | 15 | 4232.1 | 1164.7 | 2018 |
| Cubs | 173.6 | 159.1 | 129.2 | 15 | 733.9 | 449.0 | 63 |
| Younger adults | 286.2 | 376.1 | 132.6 | 15 | 4232.1 | 1014.7 | 1536 |
| Older adults | 227.3 | 322.4 | 100.9 | 15 | 3400.0 | 871.4 | 1869 |
| Aged adults | 198.5 | 412.4 | 98.9 | 15 | 4044.0 | 673.0 | 258 |
| Before roadworks | 219.9 | 303.1 | 107.7 | 15 | 4232.1 | 762.2 | 1560 |
| During roadworks | 309.5 | 407.4 | 125.9 | 15 | 3551.4 | 1190.9 | 951 |
| After roadworks | 238.1 | 357.1 | 104.6 | 15 | 4044.0 | 860.3 | 1215 |
| Adjacent | 266.8 | 385.6 | 106.5 | 15 | 4232.1 | 1132.4 | 1947 |
| Non-adjacent | 228.8 | 309.2 | 118.6 | 15 | 4044.0 | 762.2 | 1779 |

**S13 Table. Summary table of GLMM results for extra-territorial excursion distance (m).** The results for each factor level of a categorical variable are listed in order and are in comparison to the base-level of that factor. For example, the variable cohort has four factors – cub; young adult, older adult and aged. The results displayed for young adult are in comparison to cub, which is the base-level factor in this case. Similarly, each month is being compared to January, the base-level for that variable.

| GLMM with Gamma (log) distribution | | | | | | | | | | | | |  |
| --- | --- | --- | --- | --- | --- | --- | --- | --- | --- | --- | --- | --- | --- |
| Number of Observations: 3726, Groups: name: socgrp, 83; socgrp, 16; year, 7 | | | | | | | | | |  | | |  |
| *Random Effects* | Variance | Standard Deviation | | | |  |  | | | |  | |  |
| Name: socgrp | 0.68850 | 0.8298 |  | | |  |  | | | |  | |  |
| Socgrp | 0.03951 | 0.1988 |  | | |  |  | | | |  | |  |
| Year | 0.04094 | 0.2023 |  | | |  |  | | | |  | |  |
| Residual | 1.58304 | 1.2582 |  | | |  |  | | | |  | |  |
| *Fixed effects* | Estimate | Std. Error | df | | t value | | | Pr(>\|t\|) | | | |  | |
| (Intercept) | 4.43997 | 0.50304 | 8.826 | <2.00E-16 | | | | | *** | |  |  |  |
| Male | 1.06975 | 0.55515 | 1.927 | 0.053986 | | | | | . | |  |  |  |
| Younger Adult | 0.89797 | 0.48987 | 1.833 | 0.066794 | | | | | . | |  |  |  |
| Older Adult | 0.81007 | 0.49471 | 1.637 | 0.101532 | | | | |  | |  |  |  |
| Aged Adult | 1.63003 | 0.53564 | 3.043 | 0.002341 | | | | | ** | |  |  |  |
| February | 0.26617 | 0.15173 | 1.754 | 0.079387 | | | | | . | |  |  |  |
| March | -0.2224 | 0.15587 | -1.427 | 0.153641 | | | | |  | |  |  |  |
| April | -0.05724 | 0.13992 | -0.409 | 0.682469 | | | | |  | |  |  |  |
| May | -0.30231 | 0.13022 | -2.321 | 0.020261 | | | | | * | |  |  |  |
| June | -0.36674 | 0.13773 | -2.663 | 0.00775 | | | | | ** | |  |  |  |
| July | -0.48091 | 0.13479 | -3.568 | 0.00036 | | | | | *** | |  |  |  |
| August | -0.40136 | 0.13434 | -2.988 | 0.002811 | | | | | ** | |  |  |  |
| September | -0.49732 | 0.13895 | -3.579 | 0.000345 | | | | | *** | |  |  |  |
| October | -0.21395 | 0.14473 | -1.478 | 0.139345 | | | | |  | |  |  |  |
| November | 0.07935 | 0.14726 | 0.539 | 0.589985 | | | | |  | |  |  |  |
| December | -0.10046 | 0.16055 | -0.626 | 0.531501 | | | | |  | |  |  |  |
| Male: Younger Adult | -0.80781 | 0.55737 | -1.449 | 0.147247 | | | | |  | |  |  |  |
| Male: Older Adult | -0.77111 | 0.5686 | -1.356 | 0.175055 | | | | |  | |  |  |  |
| Male: Aged Adult | -1.0246 | 0.61943 | -1.654 | 0.098105 | | | | | . | |  |  |  |

**S14 Table.** **Summary statistics for frequency of extra-territorial excursions in badgers.** Means, SDs, maxima, minima and number of observations (No. obvs) for the different sexes and age cohorts, for badgers before, during and after the roadworks and for all badgers in social groups adjacent or non-adjacent to the roadworks area.

| **Group** | **Mean fETE** | **SD ±** | **Median fETE** | **Min fETE** | **Max fETE** | **No. obvs.** |
| --- | --- | --- | --- | --- | --- | --- |
| All badgers | 0.15 | 0.18 | 0.10 | 0 | 1.00 | 892 |
| Females | 0.14 | 0.16 | 0.07 | 0 | 0.90 | 463 |
| Males | 0.17 | 0.20 | 0.10 | 0 | 1.00 | 429 |
| Cubs | 0.06 | 0.11 | 0.00 | 0 | 0.47 | 46 |
| Younger adults | 0.15 | 0.18 | 0.10 | 0 | 1.00 | 379 |
| Older adults | 0.16 | 0.17 | 0.10 | 0 | 0.94 | 335 |
| Aged adults | 0.19 | 0.20 | 0.13 | 0 | 0.90 | 132 |
| Before roadworks | 0.14 | 0.17 | 0.10 | 0 | 0.94 | 400 |
| During roadworks | 0.14 | 0.17 | 0.07 | 0 | 1.00 | 261 |
| After roadworks | 0.19 | 0.19 | 0.14 | 0 | 1.00 | 231 |
| Adjacent | 0.14 | 0.16 | 0.10 | 0 | 1.00 | 504 |
| Non-adjacent | 0.14 | 0.16 | 0.10 | 0 | 1.00 | 504 |

**S15 Table. Summary table of mixed model results for frequency of extra-territorial excursions.** The results for each factor level of a categorical variable are listed in order and are in comparison to the base-level of that factor. For example, the variable cohort has four factors – cub; young adult, older adult and aged. The results displayed for young adult are in comparison to cub, which is the base-level factor in this case. Similarly, each month is being compared to January, the base-level for that variable.

| GLMM with beta-binomial distribution, response variable is a proportion formatted as successes | | | | | | | | | | | | | | | |
| --- | --- | --- | --- | --- | --- | --- | --- | --- | --- | --- | --- | --- | --- | --- | --- |
| and failures (1,0).  Number of Observations: 892, Groups: name: socgrp, 88; socgrp, 18; year, 7 | | | | | | | | | | | | |  | | |
| *Random Effects* | Variance | Standard Deviation | | | |  | |  | |  | | | |  |  |
| Name: socgrp | 0.65279 | 0.808 | |  | |  | |  | |  | | | |  |  |
| Socgrp | 0.10157 | 0.3187 | |  | |  | |  | |  | | | |  |  |
| Year | 0.06188 | 0.2488 | |  | |  | |  | |  | | | |  |  |
| *Fixed effects* | Estimate | | Std. Error | | df | | t value | | Pr(>\|t\|) | | |  | | |  |
| (Intercept) | 0.000288 | | 0.030735 | | 0.009 | | 0.99251 | |  | | |  | | |  |
| Male | 0.3309 | | 0.2061 | | 1.605 | | 0.10843 | |  | | |  | | |  |
| Younger Adult | 0.9029 | | 0.3129 | | 2.886 | | 0.003902 | | ** | | |  | | |  |
| Older Adult | 0.9222 | | 0.3269 | | 2.821 | | 0.004794 | | ** | | |  | | |  |
| Aged Adult | 1.0847 | | 0.3609 | | 3.006 | | 0.002649 | | ** | | |  | | |  |
| February | 0.7307 | | 0.2219 | | 3.293 | | 0.000992 | | *** | |  |  |  |  |  |
| March | 0.8277 | | 0.227 | | 3.647 | | 0.000266 | | *** | |  |  |  |  |  |
| April | 1.4729 | | 0.2098 | | 7.022 | | 2.19E-12 | | *** | |  |  |  |  |  |
| May | 1.6683 | | 0.1958 | | 8.521 | | <2.00E-16 | | *** | |  |  |  |  |  |
| June | 1.2767 | | 0.2005 | | 6.368 | | 1.91E-10 | | *** | |  |  |  |  |  |
| July | 1.3899 | | 0.2044 | | 6.798 | | 1.06E-11 | | *** | |  |  |  |  |  |
| August | 1.4672 | | 0.2083 | | 7.045 | | 1.86E-12 | | *** | |  |  |  |  |  |
| September | 1.7305 | | 0.2162 | | 8.006 | | 1.19E-15 | | *** | |  |  |  |  |  |
| October | 0.9088 | | 0.2157 | | 4.214 | | 2.51E-05 | | *** | |  |  |  |  |  |
| November | 0.6995 | | 0.2199 | | 3.181 | | 0.001467 | | ** | |  |  |  |  |  |
| December | 0.1845 | | 0.2325 | | 0.793 | | 0.427583 | |  | |  |  |  |  |  |
| During RW | 0.238 | | 0.1987 | | 1.198 | | 0.231094 | |  | |  |  |  |  |  |
| After RW | 0.5928 | | 0.2383 | | 2.488 | | 0.012851 | | * | |  |  |  |  |  |

**S16** **Table. Number of road crossings made by badgers before (N11), during (N11) and after (M11) construction of the new motorway.**

| **Badger Name** | **Social Group** | **Adjacent to N11/M11** | **Before** | **During** | **After** |
| --- | --- | --- | --- | --- | --- |
| Aoife | Quarry | yes | NA | 0 | 0 |
| Arthur | Bluebell Woods | yes | 0 | NA | NA |
| Beech | Bluebell Woods | no | 0 | 2 | 3 |
| Berry | Oak | yes | 1 | 1 | 0 |
| Bert | DR | yes | NA | 1 | NA |
| Billy | Quarry | yes | 0 | 0 | NA |
| Blue | Bracken | yes | 1 | NA | NA |
| Bluebell | Bluebell Woods | yes | 3 | NA | NA |
| Bob | Sycamore | yes | NA | 2 | 2 |
| Boru | Hawthorn | yes | NA | 0 | 3 |
| Brendan | The Briars | yes | 0 | NA | NA |
| Brian | The Pines | no | NA | NA | NA |
| Bruiser | Oak | yes | 0 | NA | NA |
| Bruno | The Pines | no | NA | 0 | NA |
| Buster | Oak | yes | 1 | 0 | NA |
| Carla | The Briars | yes | 4 | NA | NA |
| Cassie | Oak | yes | NA | 0 | NA |
| Cecilia | DR | yes | NA | 3 | 6 |
| Dara | Big Tree | no | NA | NA | 0 |
| Dave | Oak | yes | 0 | NA | 0 |
| Debbie | The Pines | both | 0 | NA | NA |
| Delilah | Hawthorn | yes | 0 | NA | NA |
| Dinny | Oak | yes | 15 | 2 | NA |
| Dolly | Hawthorn | yes | 6 | NA | NA |
| Douglas | Hawthorn | yes | 0 | NA | NA |
| Eddie | Bracken | yes | NA | 11 | 1 |
| Ella | Bluebell Woods | no | NA | 0 | NA |
| Ernie | Oak | yes | NA | 0 | 8 |
| Fern | Hawthorn | yes | 0 | 0 | 6 |
| Finn | Orchard | no | NA | 0 | 0 |
| George | The Briars | yes | 12 | NA | NA |
| Gina | Bracken | yes | 16 | NA | NA |
| Halle | Orchard | no | NA | 0 | NA |
| Hazel | Ballard | no | 0 | NA | NA |
| Heather | Sycamore | yes | 1 | NA | NA |
| Helene | Oak | yes | NA | NA | 1 |
| Holly | Sycamore | yes | 0 | NA | NA |
| Indy | Orchard | no | NA | 0 | NA |
| Ivy | Big Tree | yes | 0 | 1 | 0 |
| Jessie | Bracken | yes | NA | 2 | 1 |
| JJ | The Briars | yes | NA | NA | 21 |
| Juan | The Pines | both | NA | NA | NA |
| Kenny | Oak | yes | 0 | 0 | 0 |
| **Badger Name** | **Social Group** | **Adjacent to N11/M11** | **Before** | **During** | **After** |
| Kermit | Oak | yes | NA | NA | 0 |
| Kevin | DR | yes | NA | 2 | 35 |
| Lea | The Briars | yes | NA | NA | 0 |
| Leo | The Dump | yes | NA | 1 | 3 |
| Lily | Hawthorn | yes | 2 | NA | NA |
| Louis | The Pines | both | NA | 0 | 0 |
| Meg | The Pines | no | NA | 0 | NA |
| Michael | Ballard | no | NA | 1 | 1 |
| Michelle | Sycamore | no | 0 | NA | NA |
| Millie | The Pines | no | NA | 0 | 1 |
| Misty | The Pines | no | NA | NA | 0 |
| MJ | The Briars | yes | 1 | NA | NA |
| Muffin | Oak | yes | 0 | NA | NA |
| Niall | The Far Side | no | NA | NA | NA |
| O'Malley | Hawthorn | yes | NA | NA | 9 |
| Olivia | DR | both | NA | 0 | 33 |
| Oskar | Immigrant | yes | NA | NA | 0 |
| Paulie | Oak | yes | NA | NA | 0 |
| Peaches | Sycamore | no | NA | 0 | 0 |
| Ray | Bluebell Woods | both | 0 | NA | 3 |
| Romeo | The Far Side | no | 0 | NA | NA |
| Ronan | Bracken | yes | 4 | NA | NA |
| Ronnie | The Briars | yes | 1 | NA | NA |
| Rory | DR | yes | NA | 1 | NA |
| Rosie | The Lane | no | 0 | NA | NA |
| Roy | Sycamore | yes | NA | NA | NA |
| Scott | The Lodge | no | NA | 0 | 0 |
| Sheila | Oak | yes | 4 | 0 | NA |
| Snowdrop | Bluebell Woods | no | NA | NA | 0 |
| Sylvia | Ballad | no | NA | NA | 0 |
| Tiffin | Bluebell Woods | both | NA | NA | 0 |
| Tiger | DR | yes | NA | 8 | NA |
| Tyson | Ballad | no | 0 | NA | NA |
| Violet | Big Tree | no | 0 | 0 | 2 |
| Woody | Ballad | no | NA | 0 | 1 |
| Yvonne | Bracken | yes | 3 | NA | NA |
| Zia | Hawthorn | yes | 0 | NA | NA |
| **No. months in phase** | | | **41** | **22** | **14** |
| **Total no. of crossings** | | | **75** | **38** | **140** |
| **No. Crossings/Month** | | | **1.8** | **1.7** | **10** |

**S4 Fig. Sections through study area showing GPS locations before, during, and after road construction.** The green line illustrates the position of the N11 road. The blue line in the third panel illustrates the position of the M11 motorway. The red polygon shows the limit of the Compulsory Purchase Order (CPO) area within which ground clearance and road construction occurred. The exact location of the badger-proof fencing is not shown, but is between the red CPO boundary line and the motorway, often closer to the motorway itself.


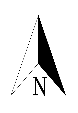

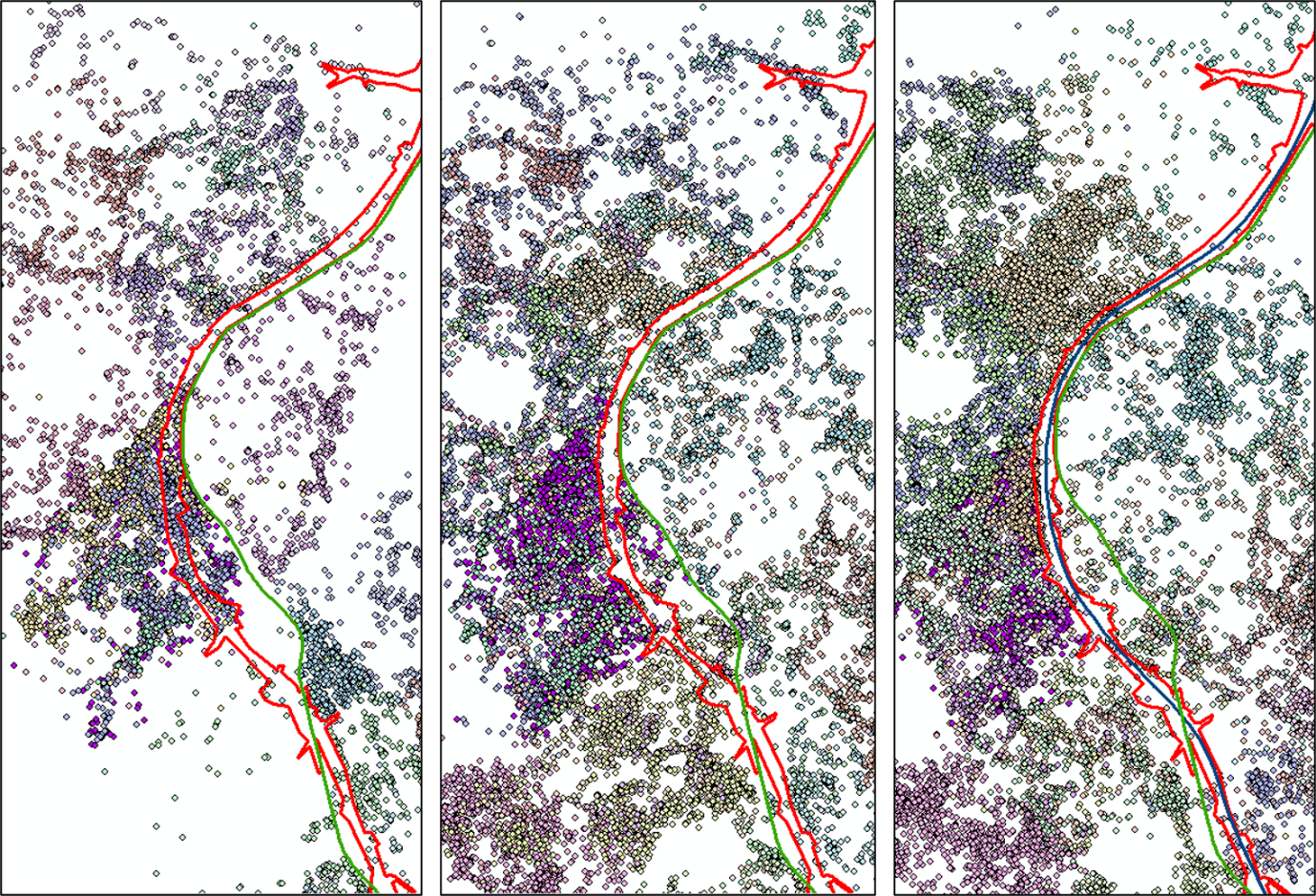


During Road Construction

Before Road Construction

After Road Construction
